# Knowledge and perceptions of Zika virus transmission in the community of Puerto Plata, Dominican Republic

**DOI:** 10.1186/s12879-019-3952-0

**Published:** 2019-04-24

**Authors:** Erik J. Nelson, Maya C. Luetke, Sina Kianersi, Erik Willis, Molly Rosenberg

**Affiliations:** 1 Department of Epidemiology and Biostatistics, Indiana University School of Public Health – Bloomington, 1025 E. 7th Street, Room C03, Bloomington, IN 47405 USA; 2 Department of Spanish and Portuguese, Indiana University –Bloomington, Bloomington, Indiana USA

**Keywords:** Zika virus, Knowledge attitude and practices, KAP, Zika prevention, Dominican Republic

## Abstract

**Background:**

Zika virus is associated with increased cases of both microcephaly and Guillain-Barré syndrome. Community knowledge, perceptions and practices to prevent infection with the Zika virus are not well understood, particularly among high risk populations living in resource-poor and Zika-endemic areas. Our objective was to assess knowledge of symptoms, health effects and prevention practices associated with Zika virus in rural communities on the northern coast of the Dominican Republic.

**Methods:**

Study participants were contacted while attending community events such as free medical clinics and invited to be interviewed regarding their knowledge, attitudes, and perceptions of Zika virus using the World Health Organization’s Zika survey tool.

**Results:**

Of the 75 Dominicans that participated, 33% did not know who could become infected with Zika. In addition, only 40% of respondents were able to identify mosquitoes or sexual transmission as the primary routes of infection though 51% of respondents thought that Zika was an important issue in their community.

**Conclusions:**

This study found that general knowledge regarding the basic risks and transmission of Zika were not well understood among a sample of rural Dominicans. Our findings highlight disparities in knowledge and perception of risk from Zika in rural areas compared to previous studies conducted in the Dominican Republic. Education about the basic risks and transmission of Zika are critically needed in these remote populations to reduce Zika transmission.

## Background

Once thought of as a mild illness, Zika virus and its potential complications have elicited a global public health campaign, with public health organizations such as the Centers for Disease Control (CDC), the Pan American Health Organization (PAHO), and the World Health Organization (WHO) coordinating efforts to minimize Zika virus risk and to prevent further spreading of infections [1, 2]. From January 2015 to September 2017, a total of 220,693 confirmed and 579,700 suspected cases of Zika have been reported in the Americas and Caribbean [3]. The WHO estimates there are currently 84 countries affected by the Zika virus, which has triggered strategic responses to prevent and manage complications of the Zika virus [4, 5]. Zika virus has taken on increased importance with the declaration of a Public Health Emergency of International Concern by the World Health Organization [6]. Due to the established clinical presentations of congenital Zika syndrome such as sever motor delay, functional impairments, microcephaly and Guillain-Barré syndrome, public health measures have focused on decreasing the risk of infection to pregnant women as well as women of childbearing age [1, 5, 7–12].

The virus has two known modes of transmission: via a mosquito vector and by sexual transmission [13–15]. The mosquito vector is thought to be the primary method for infection, though recent reports suggest that the Zika virus can survive in semen for up to 62 days after symptom onset suggesting that sexual transmission of the virus may be underestimated [6, 8, 14, 16, 17]. Accordingly, Zika infection may have long-term effects on reproductive health in addition to the neurological sequelae in newborns [18]. Sexual transmission of Zika is also concerning because sexually transmitted infections (STIs) tend to cluster geographically and occur disproportionally in areas with higher concentrations of socioeconomic disadvantage [19, 20] and limited access to sexual health resources (e.g., condoms, contraception, abortion) [21, 22]. In addition, the practice of sex tourism and partnership formation while traveling internationally (an estimated 5–50% of international travelers have sex with a new partner while abroad) raises concerns for the transmission and transportation of Zika virus to non-endemic locations, thus surveillance and preventive measures are needed in tourism hot spots—particularly in locales where prostitution is legal [23–26].

In response to the increased incidence of Zika virus infections and corresponding sequelae, the WHO released a survey of Knowledge, Attitudes and Practices (KAP) to better understand community needs and guide public health outreach [27]. Since the 2016 release of the KAP survey, it has been adapted and utilized in several Latin American and Caribbean countries [28]. Most Zika research has focused on pathophysiology, transmission, treatment and prevention. However, it is also important to understand community knowledge, perceptions and practices to determine if research findings and health education are successfully reaching at-risk populations living in Zika-endemic areas. Our objective was to evaluate the awareness, knowledge and perceived risks of Zika infection among people living on the northern coast of the Dominican Republic. This study area was selected due to the limited health education resources, high levels of poverty, Zika virus endemicity, and the high volume of tourists that frequent the area year-round.

## Methods

### Study population

**Fig. 1:**
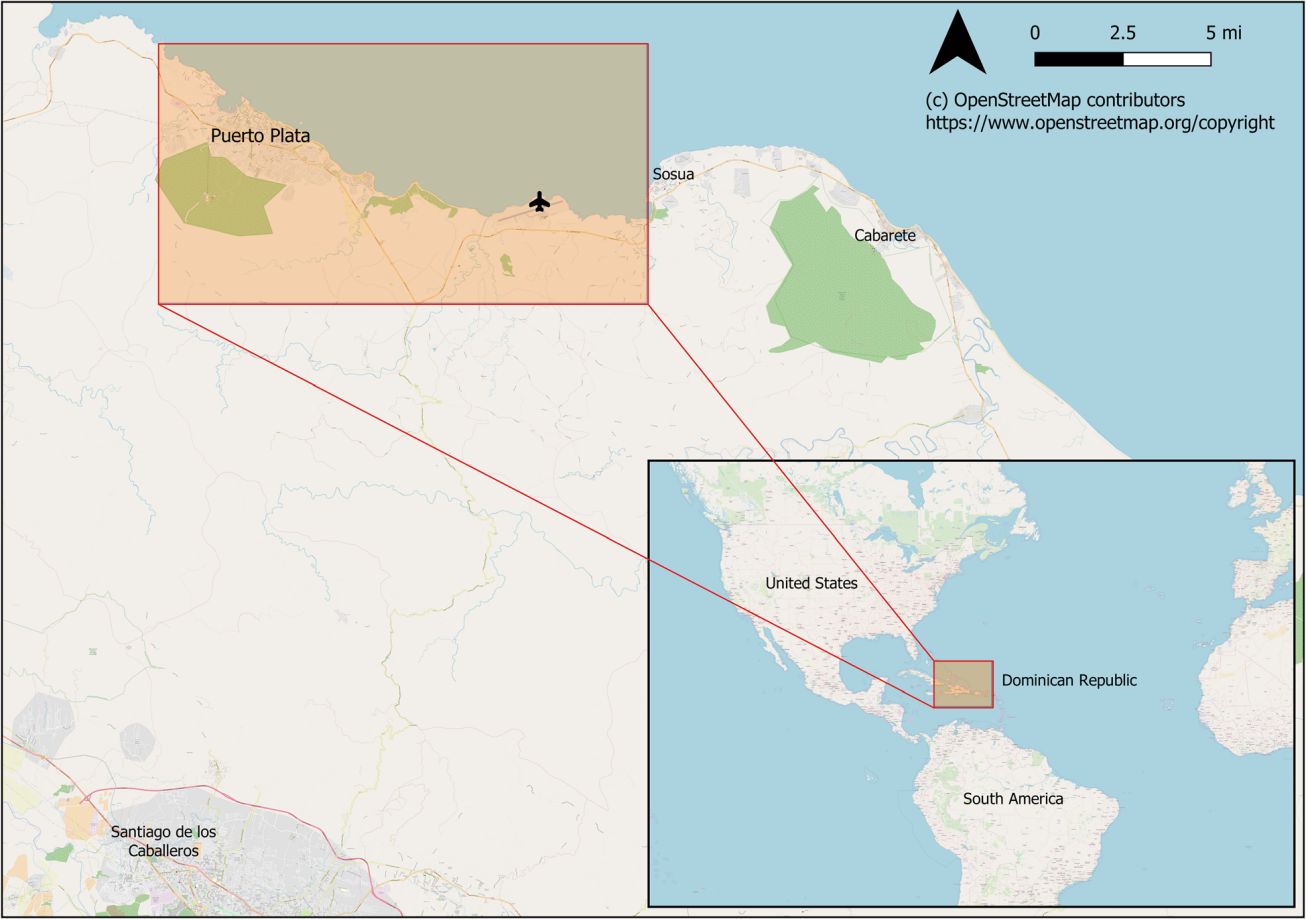
Map of the study area on the northern shore of Puerto Plata, Dominican Republic

Residents of the Dominican Republic who were aged ≥18 years who presented for free medical care or community outreach between May 10, 2017 and May 24, 2017 were eligible for this study. Potential participants were selected and approached via convenience sampling by members of the study team and invited to participate in a short survey after presenting for free optometry medical care as part of a visiting medical health outreach program or after participating in other community service outreach activities (e.g., home construction) in Puerto Plata, Dominican Republic (see Fig. 1). Approximately 10% (about 8 people) of those approached declined to participate.

### Questionnaire

After providing verbal consent, participants completed a short survey in Spanish administered by a trained study team member after they had received the aforementioned medical care or outreach services. The study team member read selected questions from the WHO’s KAP Survey to participants [27]. All surveys were conducted in private settings to facilitate genuine responses. The study team member did not read response categories to participants and allowed participants to skip questions or to respond “I don’t know” to all survey questions. The WHO KAP survey instrument is a standardized questionnaire comprised of 155 total questions, separated into four domains: knowledge, attitudes, practices and respondent demographics. The questions address both general and detailed sexual reproductive health related to Zika virus infection and practices. The survey items have been pilot tested for clarity in several languages and populations [29–33]. In order to facilitate completion of the survey, the survey was reduced to 41 questions addressing general knowledge such as signs and symptoms of infection (*n* = 5), complications and risks (*n* = 8), causes (*n* = 4), prevention (*n* = 9), trusted information sources for learning about Zika virus (*n* = 3), and demographic characteristics (*n* = 12) of the participants.

### Statistical analysis

Responses were analyzed among those who responded to each survey question using descriptive statistics to determine the frequency (percentage) of participants selecting each survey response out of the total number of participants who answered the question. Data were analyzed using R version 3.4.0.

## Results

Characteristics of the 75 study participants are presented in Table 1. More than half (64%) of the respondents were women and mean age was 42 years, with a range from 18 to 84 years. In general, respondents had low levels of formal education (52% with less than middle school educational attainment), reported low incomes [mean annual income 13,243 Dominican pesos (approximately $300 US dollars)], and were from impoverished small rural communities (known as bateyes) along the coastline.

**Table 1:** Characteristics of study participants in the Dominican Republic (*n* = 75)

	Female (*n* = 48)	Male (*n* = 27)	Total (*n* = 75)
Characteristic	n (%) or mean (SD)	n (%) or mean (SD)	
Mean Age, years	40.6 (17.4)	45.3 (19.2)	42.3 (18.1)
Age Categories			
18–29 years	17 (35.4)	7 (25.9)	24 (32.0)
30–39 years	7 (14.6)	4 (14.8)	11 (14.7)
40–49 years	8 (16.7)	5 (18.5)	13 (17.3)
50–59 years	8 (16.7)	5 (18.5)	13 (17.3)
> 60 years	8 (16.7)	6 (22.2)	14 (18.7)
Education			
Elementary School	26 (54.2)	13 (48.1)	39 (52.0)
Middle School	9 (18.8)	12 (44.4)	21 (28.0)
High School	7 (14.6)	2 (7.4)	9 (12.0)
College/University	6 (12.5)	0 (0.0)	6 (8.0)
Income, Dominican peso	8829 (10,453)	22,805 (49,120)	13,243 (29,148)

General Zika knowledge was low in this study population (Table 2). Notably, one-third of the respondents did not know who could become infected with Zika, and 40% did not know how Zika was contracted. Only 40% of respondents were able to identify mosquitoes or sexual transmission as the primary routes of infection. However, 51% of respondents thought that Zika was an important issue for their community, with the major reasons cited for concern being that they would become sick (40%), followed by babies being born with disabilities (19%), and a fear that Zika is contagious (9%). Many participants were not able to identify risks caused by Zika for babies in the womb (39%), and only 9% mentioned microcephaly.

**Table 2:** Responses to questions regarding general knowledge about Zika (*n* = 75)

	Female (*n* = 48)	Male (*n* = 27)	Total (*n* = 75)
Item^a^	n (%)	n (%)	n (%)
Who can get Zika?			
Anyone	27 (56.3)	11 (40.7)	38 (50.7)
Men	3 (6.3)	1 (3.7)	4 (5.3)
Women	6 (12.5)	1 (3.7)	7 (9.3)
Pregnant women	2 (4.2)	1 (3.7)	3 (4.0)
Boys	3 (6.3)	2 (7.4)	5 (6.7)
Girls	3 (6.3)	3 (11.1)	6 (8.0)
Don’t know	12 (25.0)	13 (48.1)	25 (33.3)
How do you get Zika?			
Mosquito bite	17 (35.4)	11 (40.7)	28 (37.3)
Sexual transmission	1 (2.1)	2 (7.4)	3 (4.0)
Virus	1 (2.1)	1 (3.7)	2 (2.7)
*Contaminated water	11 (22.9)	6 (22.2)	17 (22.7)
*Poor hygiene	6 (12.5)	2 (7.4)	8 (10.7)
*Dirty environment	4 (8.3)	2 (7.4)	6 (8.0)
*Other^b^	1 (2.1)	1 (3.7)	2 (2.7)
Don’t know	20 (41.7)	10 (37.0)	30 (40.0)
Does everybody who gets Zika show symptoms?			
*Yes	18 (37.5)	10 (37.0)	28 (37.3)
Maybe	12 (25.0)	2 (7.4)	14 (18.7)
No	10 (20.8)	10 (37.0)	20 (26.7)
Don’t know	8 (16.7)	5 (18.5)	13 (17.3)
What are the signs and symptoms of Zika?			
Fever	32 (66.7)	14 (51.9)	46 (61.3)
Headache	25 (52.1)	12(44.4)	37 (49.3)
Rash	6 (12.5)	2 (7.4)	8 (10.7)
Joint pain	16 (33.3)	6 (22.2)	22 (29.3)
Conjunctivitis (red eyes)	3 (6.3)	1 (3.7)	4 (5.3)
Diarrhea	15 (31.3)	6 (22.2)	21 (28.0)
Nausea	24 (50.0)	8 (29.6)	32 (42.7)
Hemorrhage/bleeding	2 (4.2)	1 (3.7)	3 (4.0)
Don’t know	9 (18.8)	7 (25.9)	16 (21.3)
Do you think that Zika is an important issue in your community?			
Yes	28 (58.3)	10 (37.0)	38 (50.7)
Maybe	5 (10.4)	2 (7.4)	7 (9.3)
No	15 (31.3)	11 (40.7)	26 (34.7)
Don’t know	0 (0.0)	4 (14.8)	4 (5.3)
What worries or concerns you most about Zika?			
Zika can make you sick	21(43.8)	9 (33.3)	30 (40.0)
Zika can cause babies to have disabilities	11 (22.9)	3 (11.1)	14 (18.7)
Zika can cause adults to have disabilities	2 (4.2)	4 (14.8)	6 (8.0)
Zika can be sexually transmitted	1 (2.1)	0 (0.0)	1 (1.3)
Zika will cause my child to be sick	3 (6.3)	0 (0.0)	3 (4.0)
Zika is contagious	6 (12.5)	1 (3.7)	7 (9.3)
Don’t know	6 (12.5)	10 (37.0)	16 (21.3)
If a pregnant woman has Zika, what are the risks for her fetus/baby?			
Risk of not growing or developing normally in the womb	12 (25.0)	7 (25.9)	19 (25.3)
Risk of miscarriage	19 (39.6)	5 (18.5)	24 (32.0)
Risk of being born prematurely	7 (14.6)	4 (14.8)	11 (14.7)
Risk of being stillborn	13 (27.1)	7 (25.9)	20 (26.7)
Risk of being born with microcephaly	5 (10.4)	2 (7.4)	7 (9.3)
Risk of being born with a disability	10 (20.8)	4 (14.8)	14 (18.7)
Don’t know	15 (31.3)	14 (51.9)	29 (38.7)
All pregnant women should be tested for Zika			
Strongly agree	32 (66.7)	17 (63.0)	49 (65.3)
Agree	14 (29.2)	8 (29.6)	22 (29.3)
Disagree	1 (2.1)	2 (7.4)	3 (4.0)
Strongly disagree	1 (2.1)	0 (0.0)	1 (1.3)

^a^Responses may not add up to 100% since participants could select multiple response options

^b^Other includes: larvicides (*n* = 1) and vaccinations (*n* = 1)

*Indicates a response that is contrary to fact (i.e., is not supported by scientific evidence)

Zika prevention knowledge and uptake of Zika prevention behaviors were also low (Table 3). The vast majority (88%) thought that Zika could be or ‘maybe’ could be prevented. However, 31% of participants did not know how to prevent Zika. The most commonly reported methods to prevent Zika were cleaning the household environment (33%), using mosquito nets (28%), and using mosquito repellent (13%). Over half (55%) of the participants had not taken any action to prevent Zika although they had heard of Zika. Among the 34 participants who had taken actions to prevent Zika, the most commonly reported actions were using mosquito nets (47%), using mosquito repellent (35%), and drinking clean water (27%). When asked how participants would like to receive more information about Zika, 45% reported they would like to hear directly from a medical professional and 72% reported that they would ask a medical profession directly if they had any questions about Zika.

**Table 3:** Responses to questions regarding prevention of Zika infection (*n* = 75)

Item^a^	Female (*n* = 48)	Male (*n* = 27)	Total (n = 75)
Can you prevent Zika?			
Yes	39 (81.3)	17 (63.0)	56 (74.7)
Maybe	6 (12.5)	4 (14.8)	10 (13.3)
No	2 (4.2)	2 (7.4)	4 (5.3)
Don’t know	1 (2.1)	4 (14.8)	5 (6.7)
How can you prevent Zika?			
Use mosquito repellent	6 (12.5)	4 (14.8)	10 (13.3)
Use mosquito net	10 (20.8)	11 (40.7)	21 (28.0)
Use mosquito coil/light fires to keep mosquitos away	3 (6.3)	2 (7.4)	5 (6.7)
Clean household environment	18 (37.5)	7 (25.9)	25 (33.3)
*Take medicine	6 (12.5)	3 (11.1)	9 (12.0)
*Use oral contraceptives	2 (4.2)	0 (0.0)	2 (2.7)
Don’t know	13 (27.1)	10 (37.0)	23 (30.7)
Since you heard about Zika, have you taken any action to prevent yourself from getting Zika?			
Yes	26 (54.2)	8 (29.6)	34 (45.3)
No	22 (45.8)	19 (70.4)	41 (54.7)
Since you heard about Zika, what actions have you take to prevent yourself from getting Zika?^b^			
Covered water sources	5 (19.2)	3 (37.5)	8 (23.5)
*Drank clean water	8 (30.8)	1 (12.5)	9 (26.5)
Used mosquito coil / fires to keep mosquitos away	2 (7.7)	1 (12.5)	3 (8.8)
Used mosquito nets	9 (34.6)	7 (87.5)	16 (47.1)
Used mosquito repellent	7 (26.9)	5 (62.5)	12 (35.3)
Sprayed or fumigated my home	5 (19.2)	3 (37.5)	8 (23.5)
Removed standing water	3 (11.5)	3 (37.5)	6 (17.6)
Used a condom during sex	1 (3.8)	0 (0.0)	1 (2.9)
Abstained from sex	1 (3.8)	0 (0.0)	1 (2.9)
Other actions^c^	7 (26.9)	2 (25.0)	9 (26.5)
From where/whom would you like to get more information about Zika?			
Medical professional	20 (41.7)	14 (51.9)	34 (45.3)
Family	7 (14.6)	6 (22.2)	13 (17.3)
Friends	5 (10.4)	8 (29.6)	13 (17.3)
Community leader	8 (16.7)	9 (33.3)	17 (22.7)
Television	6 (12.5)	3 (11.1)	9 (12.0)
Other media^d^	13 (27.1)	8 (29.6)	21 (28.0)
Don’t know	14 (29.2)	4 (14.8)	18 (24.0)
If you had a question about Zika, who would you ask?			
Medical professional	35 (72.9)	19 (70.4)	54 (72.0)
Family	6 (12.5)	6 (22.2)	12 (16.0)
Friends	3 (6.3)	6 (22.2)	9 (12.0)
Community leader	3 (6.3)	3 (11.1)	6 (8.0)
Don’t know	7 (14.6)	2 (7.4)	9 (12.0)

^a^Responses may not add up to 100% since participants could select multiple response options

^b^Only asked to participants who responded that they have taken an action to prevent Zika since hearing about Zika (*n* = 34)

^c^Other actions include putting screens on windows/doors (*n* = 1), washing in clean water (*n* = 5), prayer (*n* = 1), wearing covering clothes (*n* = 3)

^d^Other media includes smartphone application (*n* = 2), internet (*n* = 4), newspaper (*n* = 2), radio (*n* = 6), social media (*n* = 1), text message (*n* = 2), billboards or posters (*n* = 4)

*Indicates a preventive action that does not prevent Zika infection or transmission

## Discussion

This study found that general knowledge regarding who can contract Zika, how the virus is transmitted, and what symptoms manifest after Zika infection was low among a sample of Dominicans on the northern coast of the Dominican Republic. Our findings differed from those obtained by the Pan-American Health Organization, which used the same WHO KAP Survey to interview 608 Dominicans in and around the capital city of Santo Domingo. For example, in Santo Domingo, it was reported that approximately half of participants (51% of men and 47% of women) identified mosquitos as the cause of Zika, and two-thirds of participants (67% of men and 64% of women) were able to identify that anyone could contract Zika [34]. In contrast, in our sample, which was conducted among rural and less educated Dominicans, only 37% of participants were able to identify mosquitos as a cause of Zika and only 51% were able to correctly identify that anyone could be at risk for Zika. Furthermore, several participants reported inaccurate Zika transmission routes (contaminated water, 22.7%; poor hygiene, 10.7%; or a dirty environment, 8%) and prevention practices (e.g., 26.5% reported drinking clean water to prevent Zika infection). The observed differences in knowledge about Zika in these very different Dominican populations suggest that rural areas, where there is less accessibility to medical specialists, education and preventive tools, may be at greater risk for Zika infection. The perception of risk has a significant influence on health behavior [35–38], thus knowledge of Zika transmission mechanisms and personal infection risks are crucial for people to make the necessary behavioral changes to reduce their risk of infection.

Tourist-attracting nations, such as the Dominican Republic, could play a significant role in global transmission of the Zika virus [17, 37, 39]. For example, from January 1, 2017 to September 20, 2017, 98.5% (264/268) of Zika cases in the United States occurred in travelers returning from infected areas [40]. A survey conducted in New York in 2016 revealed that nearly one-third of pregnant women who had traveled to high-risk Zika locations were unaware of governmental travel advisories to restrict travel to these locations [41]. Other studies have also indicated low knowledge about Zika virus among travelers to Zika endemic locations [42, 43]. Hence, issuing travel advisories without effective education and notification to travelers regarding the risks, transmission pathways and prevention techniques for Zika are not sufficient alone. The need for comprehensive education and widely accessible information regarding potential disease risks, particularly for pregnant women and individuals practicing sex tourism, is needed to reduce and prevent Zika transmission in residents of the Dominican Republic and travel-acquired cases of Zika virus in non-residents.

Our findings revealed very low knowledge of the sexual transmission of Zika and consequences of infection during pregnancy in our study sample. In particular, our results differ from a study conducted among 526 Brazilian women, which found that 50.2% were aware of the sexual transmission of Zika and 98.6% knew that Zika was associated with congenital defects in newborns [44]. In the present study, only one women correctly identified that Zika could be transmitted sexually, while only 5 women (10.4%) were aware of the association between Zika and microcephaly. This finding is concerning given that the interviews were conducted several months later than those in Brazil, nearly an entire year after the CDC stated that Zika was associated with microcephaly [45] and more than 1 year after the CDC published guidelines to prevent sexual transmission of Zika [46]. Additionally, our findings show that only one person (2.9%) in our sample used condoms to prevent the spread of Zika. In a separate study conducted in rural Dominican communities, 93% of women were found to be aware of Zika but this awareness did not impact their contraceptive usage [47]. These differences indicate that there is notable variation in Zika knowledge across Dominican communities which may be a result of inaccessibility of medical services and products, such as contraceptives [47].

The majority of study participants reported being most comfortable receiving Zika-related information from medical professionals. The Dominican Republic, like many low- and middle-income countries, suffers from a shortfall of trained medical doctors. Most recent estimates indicate there are only 1.5 physicians per 1000 Dominicans [48], and residents of poor batey communities, like those enrolled in our study, are among those least likely to have to access medical care. Despite these challenges, there is precedent for successful dissemination of health information in rural Dominican Republic in the face of new disease outbreak. In 2010, a cholera epidemic began in neighboring Haiti and threatened to spread extensively across the border to the Dominican Republic [49]. Mass media messages, distribution of classroom booklets, and home visits from community health workers were largely credited with minimizing the impact of cholera in the Dominican Republic [50]. It is likely that a similarly multifaceted educational campaign would be effective at spreading accurate and actionable Zika-related health information.

This study has several limitations. First, generalizability of these study results is limited by convenience sampling among members participating in health and community outreach activities in these small rural communities. Second, there is opportunity for selection bias since this population was recruited during these communal activities and among impoverished individuals. For example, the study population may be more involved, aware, and connected to the community and therefore may have better awareness of Zika than the average community member. Furthermore, the recruited population is likely to have higher rates of poverty since they were seeking free medical care and other outreach services. Third, the small sample size of the study (*n* = 75) limited the precision with which we measured our descriptive statistics. Fourth, the KAP Survey does not capture knowledge of vertical transmission. Future studies should address knowledge and awareness of vertical transmission [51–53]. Fifth, the study data are self-reported and may be subject to recall bias. However, this study used the same survey instrument used by international agencies such as WHO and PAHO and, therefore, results can be directly compared to similar surveys conducted among various populations. Future research should be conducted in order to further illuminate the knowledge of Zika among rural Dominicans, a population that is likely to be highly affected by the virus and harder to reach by traditional means of health communication and prevention education.

## Conclusions

Measuring and increasing knowledge of Zika risks and transmission in communities, especially those where Zika is endemic, is of great public health significance. This study provides guidance for the future development of health interventions and messaging for this rural population in the Dominican Republic. Specifically, we found that information regarding the basic risks and transmission of Zika are not well understood in these impoverished and remote populations and more education and outreach are needed.

## References

[1] Centers for Disease Control and Prevention (2017). Zika Virus for Healthcare Providers.

[2] Gyawali N, Bradbury RS, Taylor-Robinson AW (2016). The global spread of Zika virus: is public and media concern justified in regions currently unaffected?. Infect Dis Poverty.

[3] Pan American Health Organization (2017). Zika cumulative cases.

[4] World Health Organization (2016). Zika virus and complications: 2016 public health emergency of international concern.

[5] World Health Organization (2017). Zika virus outbreak global response.

[6] World Health Organization (2016). Zika strategic response framework and joint operations plan.

[7] Brasil P, Pereira JP Jr., Moreira ME, et al. Zika Virus Infection in Pregnant Women in Rio de Janeiro. N Engl J Med. 2016;375(24):2321–34. 10.1056/NEJMoa1602412 PMC5323261 26943629

[8] Centers for Disease Control and Prevention (2017). Zika Virus.

[9] Counotte MJ, Egli-Gany D, Riesen M, Abraha M, Porgo TV, Wang J, Low N (2018). Zika virus infection as a cause of congenital brain abnormalities and Guillain-Barre syndrome: from systematic review to living systematic review. F1000Res.

[10] Leyser M, Marques FJP, Nascimento OJM. A multilevel-based research framework on congenital Zika syndrome. Pediatr Res. 2019. https://www.nature.com/articles/s41390-019-0349-0. 10.1038/s41390-019-0349-0 30791041

[11] Soares de Araújo JSRC, Gomes RGS, Tavares TR, Rocha dos Santos C, Assunção PMNV, de Fátima Alves Pinto D, Dantas Bezerrab BV, S dSM. Microcephaly in northeastern Brazil: a review of 16 208 births between 2012 and 2015. Bull World Health Organ. 2016. 10.2471/BLT.16.170639 PMC5096352 27821886

[12] Yuan Ling, Huang Xing-Yao, Liu Zhong-Yu, Zhang Feng, Zhu Xing-Liang, Yu Jiu-Yang, Ji Xue, Xu Yan-Peng, Li Guanghui, Li Cui, Wang Hong-Jiang, Deng Yong-Qiang, Wu Menghua, Cheng Meng-Li, Ye Qing, Xie Dong-Yang, Li Xiao-Feng, Wang Xiangxi, Shi Weifeng, Hu Baoyang, Shi Pei-Yong, Xu Zhiheng, Qin Cheng-Feng (2017). A single mutation in the prM protein of Zika virus contributes to fetal microcephaly. Science.

[13] Foy BD, Kobylinski KC, Chilson Foy JL, Blitvich BJ, Travassos da Rosa A, Haddow AD, Lanciotti RS, Tesh RB (2011). Probable non-vector-borne transmission of Zika virus, Colorado, USA. Emerg Infect Dis.

[14] Musso D, Roche C, Robin E, Nhan T, Teissier A, Cao-Lormeau V-M (2015). Potential sexual transmission of Zika virus. Emerg Infect Dis.

[15] Plourde Anna R., Bloch Evan M. (2016). A Literature Review of Zika Virus. Emerging Infectious Diseases.

[16] Harrower J, Kiedrzynski T, Baker S, Upton A, Rahnama F, Sherwood J, Huang QS, Todd A, Pulford D (2016). Sexual transmission of Zika virus and persistence in semen, New Zealand, 2016. Emerg Infect Dis.

[17] Millet JP, Montalvo T, Bueno-Mari R, Romero-Tamarit A, Prats-Uribe A, Fernandez L, Camprubi E, Del Bano L, Peracho V, Figuerola J (2017). Imported Zika virus in a European City: how to prevent local transmission?. Front Microbiol.

[18] Karwowski M. P., Nelson J. M., Staples J. E., Fischer M., Fleming-Dutra K. E., Villanueva J., Powers A. M., Mead P., Honein M. A., Moore C. A., Rasmussen S. A. (2016). Zika Virus Disease: A CDC Update for Pediatric Health Care Providers. PEDIATRICS.

[19] Beer L, Oster AM, Mattson CL, Skarbinski J, Project ftMM (2014). Disparities in HIV transmission risk among HIV-infected black and white men who have sex with men, United States, 2009. AIDS.

[20] Gindi RM, Sifakis F, Sherman SG, Towe VL, Flynn C, Zenilman JM (2011). The geography of heterosexual partnerships in Baltimore city adults. Sex Transm Dis.

[21] Rietmeijer CA, Bull SS, Ortiz CG, Leroux T, Douglas JM (1998). Patterns of general health care and STD services use among high-risk youth in Denver participating in community-based urine chlamydia screening. Sex Transm Dis.

[22] Shacham E, Nelson EJ, Schulte L, Bloomfield M, Murphy R (2016). Geographic variation in condom availability and accessibility. AIDS Behav.

[23] U.S. Department of State (2008). 2008 Country reports on human rights practices.

[24] Barzon L, Pacenti M, Berto A, Sinigaglia A, Franchin E, Lavezzo E, Brugnaro P, Palu G (2016). Isolation of infectious Zika virus from saliva and prolonged viral RNA shedding in a traveller returning from the Dominican Republic to Italy, January 2016. Euro Surveill.

[25] Duijster JW, Goorhuis A, van Genderen PJ, Visser LG, Koopmans MP, Reimerink JH, Grobusch MP, van der Eijk AA, van den Kerkhof JH, Reusken CB (2016). Zika virus infection in 18 travellers returning from Surinam and the Dominican Republic, the Netherlands, November 2015-march 2016. Infection.

[26] Workowski K, Meites E (2017). Perspectives: sex and tourism.

[27] World Health Organization (2016). Knowledge, attitudes and practice surveys Zika virus disease and potential complications.

[28] World Health Organization (2017). Mapping social science reserach for Zika virus response.

[29] Mouchtouri Varvara, Papagiannis Dimitrios, Katsioulis Antonios, Rachiotis Georgios, Dafopoulos Konstantinos, Hadjichristodoulou Christos (2017). Knowledge, Attitudes, and Practices about the Prevention of Mosquito Bites and Zika Virus Disease in Pregnant Women in Greece. International Journal of Environmental Research and Public Health.

[30] Samuel G, DiBartolo-Cordovano R, Taj I, Merriam A, Lopez JM, Torres C, Lantigua RA, Morse S, Chang BP, Gyamfi-Bannerman C (2018). A survey of the knowledge, attitudes and practices on Zika virus in new York City. BMC Public Health.

[31] Argüelles-Nava Vianey, Alvarez-Bañuelos María, Córdoba-Suárez Daniel, Sampieri Clara, Ortiz-León María, Riande-Juárez Gabriel, Montero Hilda (2018). Knowledge, Attitudes, and Practices about Zika among a University Community Located in an Endemic Zone in Mexico. International Journal of Environmental Research and Public Health.

[32] Huang Y, Xu S, Wang L, Zhao Y, Liu H, Yao D, Xu Y, Lv Q, Hao G, Xu Y (2017). Knowledge, attitudes, and practices regarding Zika: paper- and internet-based survey in Zhejiang, China. JMIR Public Health Surveill.

[33] Pooransingh S, Parasram R, Nandram N, Bhagwandeen B, Dialsingh I (2018). Zika virus disease-knowledge, attitudes and practices among pregnant women-implications for public health practice. Public Health.

[34] Pan American Health Organization (2016). Emergencia ZIKV: Resultados del proceso de consulta conocimientos, actitudes y practicas (CAP) sobre ZIKV informe de pais: Republica Dominicana. World Vision Report.

[35] Brewer NT, Chapman GB, Gibbons FX, Gerrard M, McCaul KD, Weinstein ND (2007). Meta-analysis of the relationship between risk perception and health behavior: the example of vaccination. Health Psychol.

[36] Brewer NT, Weinstein ND, Cuite CL, Herrington JE (2004). Risk perceptions and their relation to risk behavior. Ann Behav Med.

[37] Grossi PA, Percivalle E, Campanini G, Sarasini A, Premoli M, Zavattoni M, Girello A, Dalla Gasperina D, Balsamo ML, Baldanti F (2018). An autochthonous sexually transmitted Zika virus infection in Italy 2016. New Microbiol.

[38] Gujral IB, Zielinski-Gutierrez EC, LeBailly A, Nasci R (2007). Behavioral risks for West Nile virus disease, northern Colorado, 2003. Emerg Infect Dis.

[39] Ozawa H, Tajima S, Nakayama E, Kato K, Yamashita A, Sekizuka T, Kuroda M, Usuku S (2018). Isolation and complete genome sequencing of Zika virus imported from the Dominican Republic to Japan. Jpn J Infect Dis.

[40] Centers for Disease Control and Prevention (2017). Zika Virus: 2017 Case counts in the U.S.

[41] Whittemore K, Tate A, Illescas A, Saffa A, Collins A, Varma JK, Vora NM (2017). Zika virus knowledge among pregnant women who were in areas with active transmission. Emerg Infect Dis.

[42] Widmar NJ, Dominick SR, Ruple A, Tyner WE (2017). The influence of health concern on travel plans with focus on the Zika virus in 2016. Prev Med Rep.

[43] Squiers L, Herrington J, Kelly B, Bann C, Becker-Dreps S, Stamm L, Johnson M, McCormack L (2018). Zika virus prevention: U.S. Travelers' knowledge, risk perceptions, and behavioral intentions-a national survey. Am J Trop Med Hyg.

[44] Borges ALV, Moreau C, Burke A, Dos Santos OA, Chofakian CB (2018). Women's reproductive health knowledge, attitudes and practices in relation to the Zika virus outbreak in Northeast Brazil. PLoS One.

[45] Centers for Disease Control and Prevention: CDC concludes Zika causes microcephaly and other birth defects. https://www.cdc.gov/media/releases/2016/s0413-zika-microcephaly.html. Accesseed 14 Aug 2018.

[46] Oster AM, Brooks JT, Stryker JE, Kachur RE, Mead P, Pesik NT, Petersen LR (2016). Interim guidelines for prevention of sexual transmission of Zika virus - United States, 2016. MMWR Morb Mortal Wkly Rep.

[47] Shaw R, Simmons M, Nelson C, Bachelor B, Adamian S, Frausto A (2017). Unmet contraceptive needs in rural communities in the Dominican Republic during an international Zika virus outbreak. Int J Gynaecol Obstet.

[48] The World Bank (2018). Physicians (per 1,000 people).

[49] Centers for Disease Control and Prevention (2010). Update on cholera --- Haiti, Dominican Republic, and Florida, 2010. MMWR Morb Mortal Wkly Rep.

[50] Tappero JW, Tauxe RV (2011). Lessons learned during public health response to cholera epidemic in Haiti and the Dominican Republic. Emerg Infect Dis.

[51] Ciota AT, Bialosuknia SM, Ehrbar DJ, Kramer LD (2017). Vertical transmission of Zika virus by Aedes aegypti and ae. Albopictus mosquitoes. Emerg Infect Dis.

[52] Costa CFD, Silva AVD, Nascimento VAD, Souza VC, Monteiro D, Terrazas WCM, Dos Passos RA, Nascimento S, Lima JBP, Naveca FG (2018). Evidence of vertical transmission of Zika virus in field-collected eggs of Aedes aegypti in the Brazilian Amazon. PLoS Negl Trop Dis.

[53] Shi Y, Li S, Wu Q, Sun L, Zhang J, Pan N, Wang Q, Bi Y, An J, Lu X (2018). Vertical transmission of the Zika virus causes neurological disorders in mouse offspring. Sci Rep.

